# Editorial: Recent advances and perspectives on the gastrointestinal microbiota of small ruminants

**DOI:** 10.3389/fmicb.2024.1473958

**Published:** 2024-09-09

**Authors:** Einar Vargas-Bello-Pérez, Eric Altermann, Raffaella Tudisco, Qing Zhang, Anil Kumar Puniya, Anusorn Cherdthong

**Affiliations:** ^1^Facultad de Zootecnia y Ecología, Universidad Autónoma de Chihuahua, Chihuahua, Mexico; ^2^Department of Animal Sciences, School of Agriculture, Policy and Development, University of Reading, Reading, United Kingdom; ^3^School of Veterinary Science, Massey University, Palmerston North, New Zealand; ^4^Blue Barn Life Sciences Ltd., Feilding, New Zealand; ^5^Department of Veterinary Medicine and Animal Production, University of Naples Federico II, Naples, Italy; ^6^College of Forestry and Landscape Architecture, South China Agricultural University, Guangzhou, China; ^7^Dairy Microbiology Division, ICAR-National Dairy Research Institute, Karnal, Haryana, India; ^8^Tropical Feed Resources Research and Development Center (TROFREC), Department of Animal Science, Faculty of Agriculture, Khon Kaen University, Khon Kaen, Thailand

**Keywords:** rumen fermentation, microbiome, goat, sheep, diet

Recent research on the gastrointestinal (GI) microbiota of small ruminants such as goats and sheep have provided fascinating insights into their microbial ecology and its impact on health and productivity. Some key advances and perspectives in this field relate to microbial diversity and composition, revealing a diverse array of microbial species inhabiting the GI tract of small ruminants. Members of these microbiomes include bacteria, fungi, protozoa, and archaea, each playing unique roles in the nutrient digestion, immune modulation, and overall gut health. It has been demonstrated that the functional capabilities of GI microbiota, include the fermentation of dietary substrates, synthesis of vitamins, and metabolite production (e.g., short-chain fatty acids). These metabolites influence host physiology, including energy metabolism and immune function.

Recent research highlights that the GI microbiota is a key factor in improving small ruminant productivity and wellbeing. An aspect which significantly influences a small ruminant's gut microbiota composition and, hence, its functionality is the diet. For instance, the transition from forage-based to concentrate-based diets alter microbial diversity and metabolic activities, affecting animal performance and health (Xue et al.).

In contrast, understanding the microbiota-host interaction has shed light on how microbial dysbiosis contributes to diseases like gastrointestinal disorders and metabolic syndromes in small ruminants. Conversely, beneficial microbes can enhance resilience against pathogens and improve overall animal wellbeing. Improvements in high-throughput sequencing technologies (metabolomics, 16S rRNA gene sequencing, and metagenomics) have changed the way microbiota research is done in small ruminants by making it possible to comprehensively understand microbial communities and how these change with change in physiological and environmental conditions. Currently, there has been a growing interest in using probiotics and prebiotics to manipulate the gut microbiota composition in small ruminants (Mao et al.). This approach aims to enhance nutrient utilization, improve immune responses, mitigate enteric methane emissions, and reduce gastrointestinal disorders. Finally, research has also addressed the environmental impact of small ruminant farming practices on GI microbiota composition and diversity. Understanding these dynamics can lead to sustainable farming practices that optimize animal health while minimizing the environmental footprint.

Ongoing research continues to explore novel interventions and management strategies aimed at harnessing the potential of changes in diet to affect the gastrointestinal bacterial community in small ruminants. This Research Topic accepted 30 research articles covering the aforementioned aspects. In this Research Topic, studies focusing on goats (10 articles) were related to dietary fiber (Xue et al.; Zhou et al.), supplementation of sulfur amino acids (Teklebrhan and Tan), supplementation of selenium-methionine (Li L.-P. et al.), supplementation of beta-hydroxybutyrate (Chai et al.), use of dietary *Astragalus gallinaceus* (Luo et al.), the role of *Clostridium butyricum* on the intestinal microbial community (Zhang C. et al.), gastrointestinal lactic acid bacteria and their aerobic stability (Yang T. et al.), diversity of aerobic microorganisms in the rumen (Wang R. et al.), and the use of *Flammulina velutipes* mushroom residue (Long et al.).

Studies on sheep (13 articles) were focused on characterizing bacterial microbiota from different intestinal segments (Ma, Deng et al.), gut microbiome-metabolome (Ma, Yang et al.), supplementation of *Pennisetum giganteum* (Qiu et al.), immune response to Salmonella (Xu et al.), use of probiotics (Mao et al.), effect of pasture vs. pen-feed lambs on rumen bacteria (Zhang L. et al.), supplementation of grape pomace on rumen microbiome and methane production (Cheng et al.), gut microbiota of suckling lambs (Xiao et al.), effect of yeast cultures in rumen epithelium reparation (Wang H. et al.), relation between gut microbiota and growth performance (Peng et al.), supplementation of prickly ash seeds (Li D. et al.), effect of dietary alfalfa on rumen archaeal and fungal communities (Li K. et al.), and effect of *Salicornia europaea* L. on gut bacterial communities (Kamal et al.).

This Research Topic also received articles from large ruminants, such as the study of the rumen microbiome and metabolome in dairy buffaloes (Jiang et al.) and the characterization of the rumen cecum microbiome in finishing cattle (Yang Z. et al.). Also presented are *in vitro* studies, such as a comparison of the effects of rumen-protected and unprotected L-leucine on fermentation parameters, bacterial composition, and amino acid metabolism (An et al.), role of silibinin on rumen methanogenesis (Liu R. et al.), and digestive function and urea utilization ability, and bacterial compositional changes in rumen microbiota under high urea (Liu M. et al.). Another article characterized the fecal microbiota in two goat and one sheep breed distributed across sex groups (Guo et al.).

Taken together, the results from the above-mentioned studies have improved our understanding of the gastrointestinal microbiota of small ruminants. In this Research Topic, it was demonstrated that the animal's diet is one of the most practical ways to modulate the gut microbiome with concomitant changes that can improve rumen environment, immune response, growth rates, production performance, and animal health.

In this Research Topic some of the studies have demonstrated that the GI microbiome of small ruminants is involved in the breakdown of complex plant materials (Xue et al.; Zhou et al.) otherwise undigestible by the host. Microbes facilitate the plant fiber breakdown by producing enzymes like cellulases and hemicellulases, facilitating the subsequent fermentation of breakdown products into digestible compounds such as volatile fatty acids. This is important, as volatile fatty acids serve as an important energy source for the host animal and are absorbed through the gut lining into the bloodstream. Microbes in the rumen and other parts of the GI tract further contribute significantly to other metabolic processes. They synthesize essential nutrients such as vitamins (e.g., B-vitamins) and amino acids, which are critical for the host's metabolic functions. The microbiome also influences the efficiency of energy utilization from feed (Chai et al.), impacting overall growth performance (Peng et al.), reproduction, and milk production in small ruminants.

The GI microbiome interacts closely with the immune system of small ruminants (Xu et al.). Beneficial gut microbes help to maintain gut barrier integrity and promote immune tolerance, thereby reducing susceptibility to infections and inflammatory conditions. They also stimulate immune responses in the presence of pathogens, contributing to the overall resilience of the animal.

A balanced GI microbiome is crucial for preventing the disorders such as bloat, acidosis, and enteritis in small ruminants. The GI microbiome is also related to the rumen epithelium integrity (Wang H. et al.) and in this regard, the probiotics and prebiotics are increasingly used to maintain or restore a healthy microbiome balance, thereby promoting improved productive performance (Mao et al.), health outcomes, and disease resistance.

The GI microbiome of small ruminants plays a role in adapting to different environmental and dietary changes. This adaptability is important in extensive grazing systems where animals are exposed to varied vegetation and climatic conditions (Zhang L. et al.). Moreover, the composition and activity of the GI microbiome can influence the quality of products derived from small ruminants, such as meat and milk. For example, microbial metabolites (Jiang et al.) may affect composition and flavor profiles of milk.

This Research Topic clearly demonstrated that -omics and microbiome studies in small ruminants play an important role in understanding their health, productivity, and interactions with their environments. These studies have been carried out in the context of genomics to understand genetic diversity, identifying genes associated with desirable traits (e.g., disease resistance, milk production), and tracing the evolutionary history of these animals, genome-wide association studies (crucial for breeding programs aimed at improving productivity and disease resistance).

Also, transcriptomics (provides provide insights into molecular pathways and regulatory networks involved in various physiological processes), metabolomics (helps in understanding metabolic pathways, nutritional status, and metabolic responses to environmental changes or diseases (Ma et al.), and proteomics (provides insights into protein functions, interactions, and post-translational modifications, which are critical for understanding biological processes and responses) are important tools that yield specific information from GI function.

Lastly, microbiome studies can be focused on microbial community analysis or metagenomics {involves studying genetic material directly from environmental samples [e.g., feces (Guo et al.), rumen contents] to analyze microbial diversity, function, and interactions within the microbiome}.

Overall, the GI microbiome of small ruminants is integral to their digestive physiology, metabolic processes, immune function, and overall health when changes in diets occur. Research continues to uncover the complexities of microbial interactions within the gut and their implications for optimizing small ruminant production and welfare. Further research should focus on understanding the specific mechanisms through which different dietary interventions influence the gastrointestinal microbiota and associated metabolic pathways in small ruminants, to optimize health, productivity, and environmental sustainability.

